# Epidemiology of hospital-acquired infections at a tertiary care center in Lebanon

**DOI:** 10.1186/1753-6561-5-S6-P242

**Published:** 2011-06-29

**Authors:** S Kanj, G Kamel, L Alamuddin, N Zahreddine, N Sidani, ZA Kanafani

**Affiliations:** 1 Internal Medicine/Infectious Diseases, American University of Beirut, Beirut, Lebanon

## Introduction / objectives

To describe the epidemiology of hospital-acquired infections (HAI) at the American University of Beirut Medical Center (AUBMC) between October 2007 and September 2010.

## Methods

The Infection Control and Prevention Program (ICPP) at AUBMC conducts prospective targeted surveillance of device-associated infections in critical care areas (ventilator-associated pneumonia [VAP], catheter-associated urinary tract infection [CA-UTI], and catheter-related bloodstream infection [CR-BSI]). Device-associated infections are benchmarked against the rates published by the National Healthcare Safety Network (NHSN) and the International Nosocomial Infection Control Consortium (INICC). All HAIs are identified using the Centers for Disease Control and Prevention (CDC) definitions.

## Results

VAP rates were highest in the intensive care unit (ICU) (13.2-15.5/1,000 ventilator days). The most common organisms causing VAP were *A. baumanii*, *P. aeruginosa*, and *E. coli*. The respiratory care unit (RCU) had the highest rate of CA-UTI (13.6-16.0/1,000 catheter-days), with *E. coli* and *K. pneumoniae* being the most common pathogens. CR-BSI were mostly caused by coagulase-negative staphylococci, and rates ranged from 9.2 to 15.5/1,000 catheter days in ICU. The rates of device-related infections were in general higher than NHSN and comparable to INICC rates.

## Conclusion

Active surveillance remains a critical step towards recognizing and preventing hospital-acquired infections. New infection control strategies should be implemented in order to decrease the rate of device-related infections in critical care areas. These strategies include educational activities, compliance with hand hygiene and the device bundles, proper training for healthcare workers, and continuous monitoring.

## Disclosure of interest

None declared.

